# Information Spread of Emergency Events: Path Searching on Social Networks

**DOI:** 10.1155/2014/179620

**Published:** 2014-01-29

**Authors:** Weihui Dai, Hongzhi Hu, Tunan Wu, Yonghui Dai

**Affiliations:** ^1^School of Management, Fudan University, No. 220 Handan Road, Shanghai 200433, China; ^2^School of Information Management and Engineering, Shanghai University of Finance and Economics, Shanghai 200433, China

## Abstract

Emergency has attracted global attentions of government and the public, and it will easily trigger a series of serious social problems if it is not supervised effectively in the dissemination process. In the Internet world, people communicate with each other and form various virtual communities based on social networks, which lead to a complex and fast information spread pattern of emergency events. This paper collects Internet data based on data acquisition and topic detection technology, analyzes the process of information spread on social networks, describes the diffusions and impacts of that information from the perspective of random graph, and finally seeks the key paths through an improved IBF algorithm. Application cases have shown that this algorithm can search the shortest spread paths efficiently, which may help us to guide and control the information dissemination of emergency events on early warning.

## 1. Introduction

The popularity of the Internet has greatly changed people's way of life and has also changed people's political participation to a certain extent. Because of network, people's individual consciousness and active participation awareness strengthened, the choice and feedback of information have greater autonomy, and they also have more opportunity and more initiative to express their opinion. Various forms of information transmission carrier such as text, audio, and visual flooded with the Internet, which makes it difficult to distinguish fact from fiction. In addition to the rapid development of Internet technology, Internet emergencies spread fast in wide range, as well as the open network environment in dissemination, which all bring about the problems of unpredictability in spreading process and the difficulty in monitoring information dissemination. These emergencies spread throughout the Internet so that various public opinions spring out and influence widely. Therefore the supervision and management of Internet information becomes quite important.

Emergency has attracted global attentions of government and the public, and it will easily trigger a series of serious social problems if it is not supervised effectively in the dissemination process; exploring the way to prevent and solve emergency not only has become the focus of government and academia, but also has become a topic of public concern [1–3]. Therefore, the government and the online media should intervene in the emergency information's Internet dissemination timely and measurably to assure the stability and harmony of the network society. For those reasons, the study of finding hot topics, tracking the information spread, and seeking the key paths has received increasing attention and concerns from all aspects.

In the Internet world, people communicate with each other and form various virtual communities, which provide the platform of accumulation and cohesion relationships for sharing experiences. The development of Internet breaks the traditional physical limitations of nodes, through which people can build interpersonal relationships and social networks, and people can easily set up their own social ties freely using the Internet. With the development of information technology and under the background of Web 2.0, each user can have his or her own blog, BBS sites and forums, wiki, social bookmarking, and podcasts and can connect each other with the mode of Tag, RSS, IM, Email, and so forth, in virtual community. In information dissemination, any person at anywhere, at any time, can use comments in the network (such as forums, instant messaging platforms, private medias, mobile phones, text messaging, computer networks, video phone, and other personal communication technologies) and put the information share with others or express their views at the first time. In information dissemination and comment, the multimedia, interactivity, and other characteristics of network have accelerated information spread and can easily arouse widespread public concern and influence. According to the theory of six-degree separation [4], more individuals gradually become the dissemination nodes of emergency information, which form a large social network as the specific information spread system on the Internet. Individuals can easily find channels to disseminate information and gradually become more and more emergency information dissemination node.

## 2. Related Work

Since the anthropologist Barnes firstly came up with the concept of “social network” to analyze the social structure of a fishing village in Norway, social network analysis is considered to be one of clear and convincing perspectives that are used in the study of social structure, and the perspective of social relationship has better explanation than the perspective of individual attribute. However, the virtual community connected by social networks on the Internet is a different social entity from real society in people's communication actions and information spread paths [5], and this may lead to a complex diffusion pattern of public opinions in emergency events [2, 6].

The influence of public opinion in the Internet has both positive and negative aspects [7]. Many established some models to test frame building and frame setting between online public opinion and media coverage, used a methodological model to test the frame building, and found that online public opinion plays an important role in transforming the original local event into a nationally prominent issue and also exerts a significant frame-building impact on subsequent media reports but only in the early stage of coverage. Some studied public opinion polls in different periods and found complex changes of public opinion in a continuous period of time [1, 8].

Topic detection technology is a kind of text classification and clustering [9, 10]. After mining and analyzing the regular pattern of users visiting BBS, also mining the posts by ARC-BC text classification algorithm on BBS, results showed that certain users and the collective performance have the obvious similarity or difference. It indicated that this algorithm presented a good performance on BBS text classification [11]. In some ways, topic tracking is very similar to a filtering task in information retrieval [11–13]. The algorithm on Internet data discovery and dissemination is relatively mature, but there are less systematic topic discovery applications.

From a methodological point of view, social network analysis technologically describes the relationship mode between actors under certain circumstances using random graph theory, which was proposed by Erdös and Rnyi systematically in 1960 [14] and has expanded rapidly from then on. We first construct the scale-free networks following the same method used in the literature [15] by Albert et al. Starting with a small number of vertices (or nodes), a new vertex with *m* edges (or links) is added at each time step. In the literature [15], it has been shown that the above method of constructing networks, which is composed of ideas of growth and preferential attachment, results in the so-called scale-free networks, which shows the power law behavior in the connectivity distribution.

Based on social network theory and communication theory, the information spread of the emergencies has its corresponding diffusion process and spread path; some key nodes of information spread play the roles of the “amplifier” in all kinds of information spread process. So we should find out the key nodes and key spread paths to guide the normal dissemination of emergency information and control distorted information in spread process, which is very important for us. The key paths problem is one of the most fundamental optimization problems in network. Algorithms for this problem have been studied for several years [16–19] and get fruitful research results. A suitable implementation of these algorithms appears in [20]. In this paper, we apply the improved Intersect-Bellman-Ford (IBF) algorithm [20] and propose new solutions to explore the key information spread paths of emergency events on Internet based on social networks. The application of this algorithm is based on experimental data [21].

## 3. Technique for Topic Detection and Topic Tracking

### 3.1. Topic Detection Technique

As to topic detection, we present a way to retrieve keywords that user concerned by search engine or a website search, through the collection of keyword search results list to reduce range of acquisition; besides, the collected data are those related to users interested keywords which all guaranteed the accurate collection. API is a call-level interface of a system application that executes an application program command. Internet search engine of API is a kind of search service to provide service for users to invoke its services conveniently.

Data acquisition part is divided into search engine and forum these two blocks to retrieve data, mostly because the search engine has all indexed data on the Internet; it is of much higher efficiency by using the services provided by the search engine, but the search engine return data contains most of the Internet site types except for forum because of its large number, high update speed, and other characteristics, so in these two ways, it can cover almost all data on Internet. Data exchange is mainly responsible for the external collection, in response to search engine and objective forum, respectively, by different data acquisition methods: the entry of data collection is target keywords, which can be manually or automatically detected as input data. Data exchange was completed in the external data source interface, such as search engine API debugging and specific forum crawling. At the same time, the retrieved and collected data will be cleaned and stored into the database specification through data exchange.

**Figure 1:**
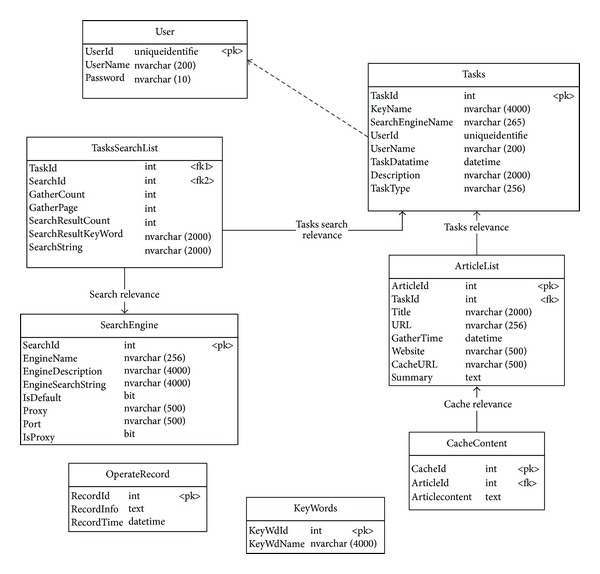
Database structure.

Because the methods of Internet data acquisition are usually different, we need to set information associated with the search engine, namely, Search Engine table, to manage search engine which obtains data through API. Every time the information collection is a collection task, in response to a search engine, and forums are required to manage the information acquisition task, namely, through the tasks table that manages each task. because each time the data acquisition task is initiated by keyword which is managed by special keyword table.  Figure 1 shows the database structure.

### 3.2. Topic Tracking Algorithm

Topic tracking is subtask of TDT; its purpose is to identify and monitor related subsequent reports of several given topics. Topic tracking can help people collect and organize scattered information effectively, which focuses primarily on the discovery of related follow-up information, and collect information locally for further analysis. In this way, we may get an overall understanding of full details. Topic tracking process is a follow-up process of topic detection, which is included in the topic discovery process.

Topic tracking can also be understood as to search its related or similar points to the latest information with limited features, to collect qualified information locally, recalculate the topic feature, and then to search in the Internet with new features, so repeatedly, in order to detect all relevant reports and update coverage of the objective topic.

Topic tracking model is a description model to be provided to user on information spread intuitively. Due to the particularity of Internet data, the traditional spread model cannot be applied to Internet tracking field completely; it is necessary to design a topic tracking model in line with the Internet data characteristics. The key to Internet information spread is the time of publication and publishing site. So we need to obtain all publication times and websites of topic and then rank according to time relevance to get detailed description of dissemination.

In this paper, we calculate the correlation degree of the article on the same topic according to the topic relevance degree based on time, give spread relationship from different sites under the same topic in the form of spread net, analyze information dissemination tendency combined with attention degree, and present propagation graph to the user.

## 4. Virtual Communities and Social Network

Social network structure determines the relationship between actors. It can be described by using kind of quantitative languages with the network data. Based on this theory, we can understand attributes of actors and the whole network according to the relationship modes between actors from the structural context. Like real communities, social networks in virtual communities also have similar interpersonal network relation characteristics, such as strong ties and weak ties and; users can freely exchange information, share knowledge, and get social support in virtual communities, and all of these are the basic platform for information diffusion and dissemination of an emergency event. It is the formalized definition by using points and lines to represent social network.

In order to explain the utility of social networks, we consider each individual spreader as an independent ontology. In emergency events, the most efficient method of information spread to the end user is to find the shortest spread path. So the user and the relation between different users in virtual communities must be abstracted as a graph structure by using node and edge, called constructing network topology in the geographic information system. We begin by specifying the notation that will be used in the rest of this Paper. The graph is defined as follows.

Everyone is in one or several groups; the definition of a graph can be described in various systems. However, in this paper, each person is expressed as one vertex in the graph. We defined their relation to those people who have “relationships” as “acquaintance,” which is described as a connection between two nodes in the network graph.

We only consider the graphs that comply with the following restrictions.

1. Undirected: each edge does not show the inherent direction.
2. Unweighted: each edge has no weighted value.
3. Simple: it is not possible to use multiple edges to connect the same pair of vertices, and the vertex cannot be connected to itself.
4. Sparse: for undirected graph, when the largest scale *m* = *E*(*G*) = *C*_*n*_^2^ = *n*(*n* − 1)/2, the undirected graph is changed to a fully connected graph; it also can be called complete graph. Sparse means *m* ≤ *n*(*n* − 1)/2,   which is equivalent to *k* ≤ *n*.
5. Interconnected: any vertex can reach any other vertex by paths which are constituted by limited number of edges.

The definitions of graphs and the related characteristics are given as follows [20].

**Definition 1**

Let *G* be a graph composed by the connection between points. *V*(*G*) denotes the vertex set of graph *G*, and *dv* is the degree of vertex *v*.  *E*(*G*) denotes the set of edges. Edge (*v*, *w*) is the connection between vertex *v* and vertex *w*.

Let graph be expressed by adjacent matrix. If there is an edge between vertex pair (*v*, *w*), then *A*_*v*,*w*_ = 1; else *A*_*v*,*w*_ = 0. We use convergence rate *C* and average distance *L* to express graph's character.

**Definition 2**

*l*(*v*, *w*) means the minimum number of edges from vertex *v* to vertex *w*, which is also known as the shortest path length between vertices *v* and *w*.  *L*(*G*) is the average distance between every two vertices:

(1) $$\begin{array}{c} L\left(G\right)=\frac{1}{n}\sum l\left(v,w\right). \end{array}$$

**Definition 3**

Let *V*(*G*) = {1, 2,…, *n*} be a ground set of *n* elements; *G*(*n*, *M*) is a labeled graph of the vertex set = {*l*, 2,…, *n*}  and  *M* is the number of randomly selected sides. *G*(*n*, *M*) can be abbreviated as *G*_*m*_.

A closely related model, denoted by, for example, *G*_*n*,*p*_, where 0 ≤ *P* ≤ 1, is obtained by taking the same vertex set but now selecting every possible edge with probability  *P*, independently of all other edges. We are mainly interested in the case where the number of vertices is very large and especially asymptotic when *n*, and *P* is a given function of *n*.

**Definition 4**

Suppose that a random regular undirected graph *G*, which has *n* vertices, for every vertex has *l* edges link with other *l* vertices, in which *l* vertices are selected randomly with a fixed probability; the convergence rate *C* of this random graph is the average value of convergence rate *C*_*i*_, where

(2) $$\begin{array}{c} C_{i}=\frac{\sum_{1\leq j,k\leq n,j\neq k}^{}A_{i,j}A_{j,k}A_{k,i}}{l\left(l-1\right)}. \end{array}$$

## 5. Algorithm for Searching the Information Spread

We first study the communication nodes and the key paths of network and describe these issues as an intelligent routing problem on information spread and diffusion of emergency events. Each disseminator or agent considered as a “bridge” is called network node. In order to achieve the desired effect, we need to collect each agent's preferences, associated social information, and other streaming data, and we also need to analyze and mine congestion status of communication channels, such as blog and BBS, the special destination of each network, and the best routing and direction of the network by specific algorithm; these results are in information table form of each routing selector.

### 5.1. The Shortest Path Finding Algorithm

**Figure:**
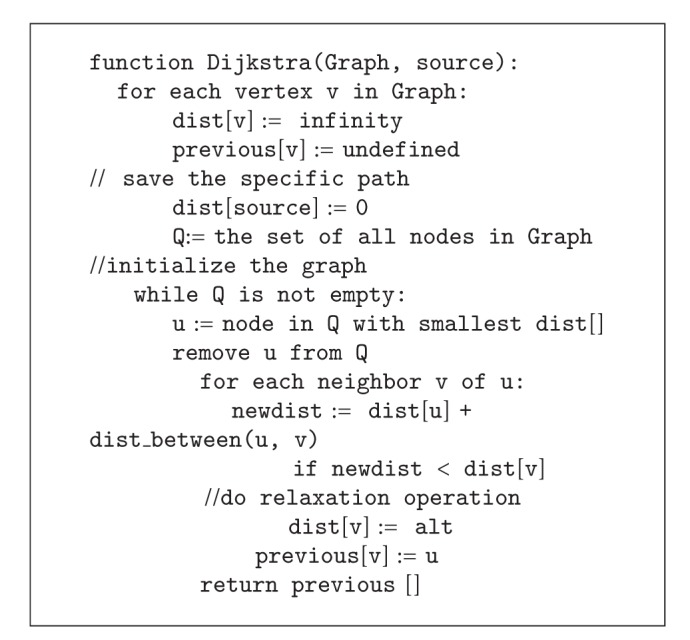
Pseudocode 1

This paper highlights the results obtained by the random graph; these results are in accordance with the rapid direct path finding algorithm after pretreatment. Without loss of generality, we assume that a single inquiry routing diffusion time is subjected to *O*(*V*log⁡*V*) [20].

Dijkstra algorithm [22] is often used in solving the problem of the shortest spread path routing between any two points *x*,  *y*. Although an emergent event's routing may not necessarily be the optimal solution, if the suboptimal solution has high efficiency and its accuracy is passable, it can be acceptable. In order to reduce the searching range and improve processing speed, many improved algorithms such as two-way Dijkstra algorithm are good choices. Routing algorithm's problem domains are as follows: the algorithm should give an optimal path from starting point to the destination of a given network emergencies in the information map of related persons and guarantee the minimum cost from the starting point to the destination.

According to [22], the modified Dijkstra's pseudocode is shown in Pseudocode 1. It is easy to find that, if we use an array or list to maintain a priority queue in this algorithm, the time complexity of each inner loop execution is the fact that the out layer network needs to be performed *d*||*V*||^2^ times, so the total time complexity is *O*||*V*||^2^ in the realization. Then the rest of the question to be considered is how to find key path in the emergency information spreading.

### 5.2. The Improved IBF Algorithms

Since the intersection-centric route guidance architecture refers to algorithm's modification of the distance, we solve the problem with a new algorithm according to the literature [20]. They proposed an improved IBF algorithm which is intersection centric and considered the character of *N* regular random graph.

The precondition of Intersect-Bellman-Ford algorithm is that the agent who acts as “bridge” prefers to collect information of emergency, or we call him/her gossip person in reality like routers that send Hello message in network and then find updated information from upper reaches. So every vertex has enough neighbors' information. The improved IBF algorithm is conducive to promote the performance of system; the application of this algorithm is expressed in [20].

If some communication channel is blocked, the calculation results of improved IBF will be changed by the changing of the numerical distance of two neighbors in network. If the path is impassable, we can set uv.weight into infinity. When there are many sudden emergencies, the agent who acts as bridge needs only to give a network link to the next node, as long as the emergency source website is not removed, and then there will not be a great influence on the entire communication system.

## 6. Application Cases

### 6.1. Case A

In order to test the efficiency and accuracy of topic detection and topic tracking, we choose a typical emergency event to do the experiment: an in construction building collapse occurred in Shanghai, which triggered a great discussion by the public.

On June 27, 2009, a 13-layer in construction building collapse in Shanghai Lotus Riverside, killed one worker. On July 3, findings of investigation by Shanghai City Hall reported that the main cause of collapse is the pressure difference on both sides of building; netizens have questioned about that; and then 7 of the relevant responsibility persons were arrested in accordance with the law; the clean-up work and housing compensation matters began on August 5.

At the beginning, netizens only had criticism on the real estate developers but not on the government level. But when Shanghai City Hall published their findings that the collapse event is due to the pressure difference, it has aroused awidely query and caused a great disturbance.

According to the previously mentioned algorithm, the experiment is divided into three steps.

1. Capture the initial release site of the original network emergencies, including structure and text.
2. Find out how other users get the release source of network emergencies based on publication time, whether from the URL site, the reminder corners of the site, or the dialog box, and so on.
3. Confirm whether someone is affected by the information based on his/her comment on the forum and blog related to the topic. The small number may cause the crawled network size to be too small. Therefore, it is necessary to select an appropriate and effective starting point.

**Figure 2:**
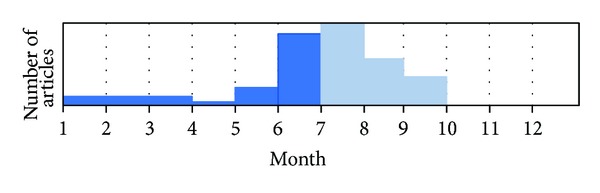
The number of articles in the year of 2009.

In addition, in order to understand the information spread of emergencies intuitively, this paper designed multiple start origins experiment. To the same point, we carry out web crawling twice, one is used the content relevance algorithms, while the other is not used.Figure 2 is the number of articles about this event in the year of 2009.

We can see clearly that when the collapse events occurred in June, the articles of discussion and communication had a rapid increase in July and August and then leveled off in the following months. The classification of search source form is in Table 1.

### 6.2. Case B

In order to verify the correctness of social networks theory and random graph theory used in this paper and reflect the spread characteristics of network emergencies more intuitively, according to the previously mentioned algorithm, we designed relevant experiments. The experiment is divided into three steps.

1. Mine the social network from the original Internet network, including the structure and text.
2. Analyze the potential communication channels and their structural analysis.
3. Find and streamline the spread key path.

The choice of start site emergency will largely determine the end site and dissemination process. For example, if the crawled number of published articles of the related emergency is small, it may cause the network scale to be too small, so we must choose a suitable and effective starting point or multiple starting points and origins. For this reason, we carefully selected a seed site, which at least meets the following two requirements: large amount of published articles and update frequently and being rich in article comments and relative links.

We also measure Internet users' active level by the number of the responses, discussions, and comments when an emergency event occurred, while the URL mail, video, searches, the portal site, bulletin board, and partition reminding corners of this event are the dissemination channels. To verify the feasibility of network spread algorithm mentioned in the last section, we will take the case of “Zhu Ying Qing Tong” to analyze the detail process of experiment's validation.

Zhu Ying Qing Tong, when she updated her photos in real time in her personal blog on Tianya virtual community, the click-through rate of her personal blog soared to 13,000 within a month and the peak click-through rate was more than hundreds of thousands per day. Such high click rate was stunning; its popularity index is enough to shame the early network literature writers, just like the author of “the first contact.” According to Hangzhou “e Time Weekly” said that, up to 5:30 pm on February 19, Zhu Ying Qing Tong's blog visitors are more than 940,000 within two days; the number is approaching one million and exceeds the historical record of personal blog visit rate. The “e Time Weekly” is the first magazine that reports the event of Zhu Ying Qing Tong. On Zhu Ying Qing Tong Memorabilia of her personal website, there are article titles provided by herself with enough sensational: “I took off clothes in front of people,” “why should I feel shame,” and so on. Zhu Ying Qing Tong also releases her new articles in Sina blog: http://blog.sina.com.cn/zyqt and her official website “Women Road Network”: http://zyqt.blog.sohu.com/. People who respond to her articles are the ones who were impacted, and the man who forwards these links is the agent.

**Figure 3:**
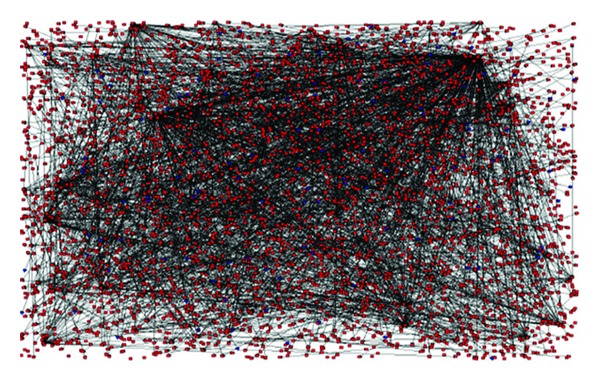
Original network of information spread.

**Figure 4:**
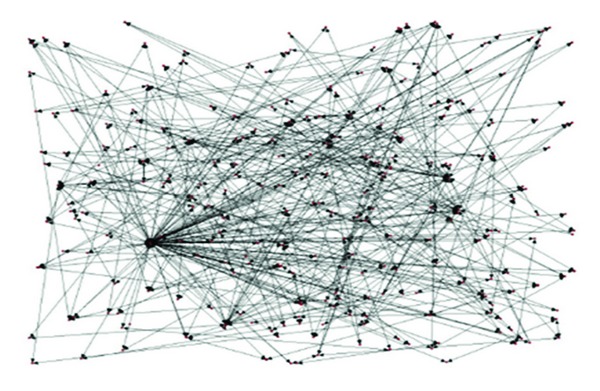
The shortest spread paths of information spread.

In accordance with our experiments step, we capture the initial release point of this information, firstly, and then find out how other people get the information, whether from to the URL site of the release source, the reminder corners of the site, or the dialog box, and so on; we finally confirm who is the last one affected by the comment on the forum or his/her blog. The original information spread is shown in Figure 3, and the key spread path that is trimmed by the algorithm can be seen in Figure 4 [21].

From the comparison of these two figures, we can verify that the algorithm has solved the problem of searching the shortest spread paths efficiently.

## 7. Conclusion

The information spread of emergency events has the characteristics of quick speed and wide range on the Internet, and the network environment is quite loose, so it is of great difficulty to supervise and control the spread process of Internet emergencies but meanwhile of great importance [23].

In this paper, we analyze the process of information spread on Internet, abstract the user and the relationship in virtual communities as a graph structure by use of node and edge, describe the information diffusion, dissemination, and the impacts of emergency events from the perspective of random graph, and then seek the key paths through Dijkstra algorithm and an improved IBF algorithm. By taking the application cases to analyze the detail process of experiment's validation, we can find that the algorithm has solved the problem of searching the shortest spread paths efficiently.

## Figures and Tables

**Table 1:** Search source classification.

Website (rank by number)	Number of articles
Baidu Forum	2381
Sina Forum	1879
Souhu Community	1765
Tianya Community	1157
Tom BBS	987
